# Task-dependent and distinct roles of the temporoparietal junction and inferior frontal cortex in the control of imitation

**DOI:** 10.1093/scan/nsu148

**Published:** 2014-12-05

**Authors:** Jeremy Hogeveen, Sukhvinder S. Obhi, Michael J. Banissy, Idalmis Santiesteban, Clare Press, Caroline Catmur, Geoffrey Bird

**Affiliations:** ^1^Cognitive Neuroscience Laboratory, Rehabilitation Institute of Chicago and ^2^Department of Physical Medicine and Rehabilitation, Feinberg School of Medicine, Northwestern University, Chicago, IL, USA, ^3^Department of Psychology, Neuroscience & Behaviour, McMaster University, Hamilton, ON, Canada, ^4^Department of Psychology, Goldsmiths and ^5^Department of Psychological Sciences, Birkbeck College, University of London, London, UK, ^6^Department of Psychology, University of Surrey, Guildford, UK, and ^7^MRC Social, Genetic, and Developmental Psychology Centre, King’s College London and ^8^Institute of Cognitive Neuroscience, University College London, London, UK

**Keywords:** imitation, mimicry, mirror system, transcranial direct current stimulation, temporoparietal junction, inferior frontal cortex

## Abstract

The control of neurological networks supporting social cognition is crucially important for social interaction. In particular, the control of imitation is directly linked to interaction quality, with impairments associated with disorders characterized by social difficulties. Previous work suggests inferior frontal cortex (IFC) and the temporoparietal junction (TPJ) are involved in controlling imitation, but the functional roles of these areas remain unclear. Here, transcranial direct current stimulation (tDCS) was used to enhance cortical excitability at IFC and the TPJ prior to the completion of three tasks: (i) a naturalistic social interaction during which increased imitation is known to improve rapport, (ii) a choice reaction time task in which imitation needs to be inhibited for successful performance and (iii) a non-imitative control task. Relative to sham stimulation, stimulating IFC improved the context-dependent control of imitation—participants imitated more during the social interaction and less during the imitation inhibition task. In contrast, stimulating the TPJ reduced imitation in the inhibition task without affecting imitation during social interaction. Neither stimulation site affected the non-imitative control task. These data support a model in which IFC modulates imitation directly according to task demands, whereas TPJ controls task-appropriate shifts in attention toward representation of the self or the other, indirectly impacting upon imitation.

## INTRODUCTION

The importance of socio-cognitive ability for human health (Kiecolt-Glaser *et al.*, 1984; Cohen, 1988), wealth (Lopes *et al.*, 2006; Silk, 2007) and happiness (George *et al.*, 1989; Kaufman *et al.*, 2004) is now well-established. However, it is only recently that the importance of the top-down control of socio-cognitive networks has been realized (Frith and Frith, 2006; Satpute and Lieberman, 2006; Spengler *et al.*, 2009; Cook *et al.*, 2012). For example, despite the fact that the general tendency to imitate the posture (Lafrance and Broadbent, 1976), facial expressions (Neal and Chartrand, 2011) and actions (Chartrand and Bargh, 1999) of our interaction partners leads to high quality social interaction (Lakin and Chartrand, 2003), imitators dynamically modulate the degree to which they mimic others as a function of variables such as power relationships, group dynamics and relationship quality (Chartrand and Lakin, 2013). The importance of top-down control of socio-cognitive processes is evidenced by the severe social deficits seen in autism spectrum disorders (ASDs) when this control goes awry (Bird *et al.*, 2006; Cook and Bird, 2012), and by the fact that, at least in the case of imitation, control of social cognition relies on a dedicated neural network, independent of the standard cognitive control network used to inhibit or enhance other automatic behavioral tendencies (Brass *et al.*, 2005; Wang and Hamilton, 2012).

The ability to imitate the actions of others is thought to be mediated by the human mirror neuron system (MNS), comprising portions of the inferior frontal cortex (IFC) and parietal cortex (Iacoboni *et al.*, 1999; Decety *et al.*, 2002; Heiser *et al.*, 2003; Chaminade *et al.*, 2005; Catmur *et al.*, 2009). However, mirror neuron activity does not always produce imitation, and functional magnetic resonance imaging (fMRI) and brain stimulation studies suggest that the top-down control of the mirror system is accomplished via a network of regions including regions of the IFC (co-located with those involved in mirroring, but extending more anteriorly^1^; Brass *et al.*, 2005; Catmur *et al.*, 2009, 2011), the temporoparietal junction (TPJ; Brass *et al.*, 2005, 2009; Santiesteban *et al.*, 2012a; Sowden and Catmur, 2013) and medial prefrontal cortex (mPFC; Brass *et al.*, 2005, 2009; Wang and Hamilton, in press; Wang *et al.*, 2011). Networks mediating the control of imitation show functional and partial anatomical overlap with those supporting Theory of Mind (ToM; the ability to represent the mental states of oneself and others). For example, both the TPJ and mPFC are reliably activated in neuroimaging studies of ToM (Castelli *et al.*, 2000; Frith and Frith, 2003; Mitchell, 2008; Van Overwalle, 2009; Zaki *et al.*, 2010). Further, functional relationships between imitative control and ToM have been demonstrated, whereby training participants to control imitation improves their ability to take another’s visual perspective (Santiesteban *et al.*, 2012b), while an impaired ability to control imitation is correlated with reduced ToM ability in individuals with ASD (Spengler *et al.*, 2010a) and in patients with lesions to mPFC or TPJ (Spengler *et al.*, 2010b).

Findings such as these have prompted the suggestion that the core neurocognitive function of TPJ and mPFC in the social domain is to control the degree to which the self or another is represented (Brass *et al.*, 2005, 2009). It is argued that when there is a task-relevant conflict between the motor plans, perspectives or knowledge of the self and the other, or when it is easy to confuse representations of the self and other (for example, when two people perform a synchronous action), then control of self and other representations is required for successful task performance. The control of self and other representations would impact upon both the degree of imitation—by either boosting the representation of another’s action (increasing imitation) or one’s own motor plan (decreasing imitation). Controlling self and other representations would also impact upon ToM—by governing the extent to which one’s own or another’s, mental state is represented and/or attended to. Therefore, the TPJ and mPFC are likely to be indirectly involved in controlling imitation through controlling the activation of self vs other representations.

In contrast to the indirect role of TPJ and mPFC, the IFC appears to be more directly involved in controlling imitation. Previously, IFC has been causally linked to both the performance of imitative acts (Iacoboni *et al.*, 1999; Heiser *et al.*, 2003) and their inhibition (Brass *et al.*, 2005; Catmur *et al.*, 2009). It is our contention that, in addition to mirror neurons in IFC, a complementary set of cells act as a ‘gain control’ on imitation, increasing or decreasing the influence of the mirror system upon behavior (cf. Kraskov *et al.*, 2009; Mukamel *et al.*, 2010). Under this view, the nodes of the imitation control network perform distinct functions: TPJ and mPFC control self-other representations with an indirect impact upon imitation, while IFC has a direct impact on the degree of imitation.

**Fig. 1:**
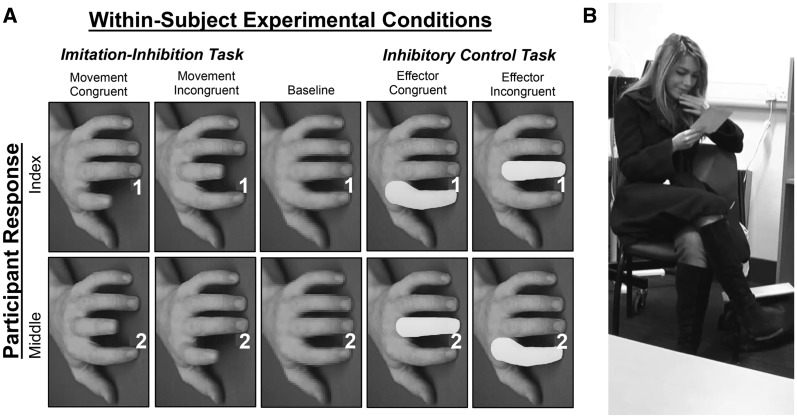
(**A**) Final frames of each trial in the imitation inhibition and inhibitory control tasks as well as the baseline condition. Note: Font size of the numerical cues is exaggerated for display purposes. Participants were instructed to respond with an index finger lift in response to the presentation of a 1 and a middle finger lift in response to presentation of a 2. (**B**) During the social interaction the confederate repeatedly touched her face. The degree of imitative behavior exhibited by the participant was analyzed as a function of brain stimulation.

To investigate the possible dissociation between direct and indirect control of the mirror system, we used anodal transcranial direct current stimulation (tDCS) to enhance cortical excitability at IFC and TPJ, two components of the imitation control network. Following stimulation, participants completed three tasks. On critical trials of the first task—referred to here as the imitation inhibition task—participants performed finger movements in response to a symbolic cue while observing finger movements which were incongruent with their response (Figure 1A). Thus, participants were required to enhance representation of their own motor intention and suppress that of the other in order to inhibit task-inappropriate imitation (Santiesteban *et al.*, 2012a; De Coster *et al.*, 2013). On critical trials of the second task—referred to as the non-imitative inhibitory control task—participants were required to perform finger movements in response to a symbolic cue while observing a second, non-action, cue that was incongruent with their response (Figure 1A). This task therefore assessed the degree to which any effect of stimulation observed on the imitation inhibition task was domain-general or specific to the control of imitation. A third task—referred to here as the social interaction task—required participants to engage in a social interaction with a confederate who repeatedly performed a target behavior (i.e. face touching). In this context, individuals have been shown to increase the degree to which they imitate in order to promote high-quality social interaction.

Thus, the non-imitative inhibitory control task is distinguished from the two tasks requiring imitative control by the lack of an observed movement performed by another agent. The two imitative tasks are distinguished by two key factors. The first factor concerns the direction of imitative control required: the imitation inhibition task requires imitation to be down-regulated, while during the social interaction task an increased level of imitation leads to better performance. The inclusion of these tasks therefore allows task-dependent effects of stimulation to be observed. The second factor concerns the degree to which the tasks require a resolution of conflicting self- and other-related motor representations. This ‘self-other control’ requirement is central to performing the imitation inhibition task successfully as the incongruent motor plan of the other must be suppressed in order to carry out one’s own motor plan. In comparison, the requirement for self-other control in the social interaction task is minimal: participants’ actions are not synchronous with those of the confederate (a few seconds delay is necessary to prevent the imitative behavior from entering into conscious awareness; Bailenson *et al.*, 2004; Bailenson and Yee, 2005), and therefore there is little opportunity to confuse agency. In addition, there is no requirement for the participant to keep separate the representations of their own actions and those of the confederate in order to inhibit imitation as imitation leads to positive outcomes on this task (Figure 1B). Thus, if the TPJ serves to control representations of the self and other, then an effect of TPJ stimulation is expected on the imitation inhibition task but not on the other tasks. If IFC exerts a direct effect on the mirror system in order to control imitation according to task demands, then it is expected that stimulation of IFC should increase the level of imitation in the social mimicry task, but decrease the level of imitation in the imitation inhibition task.

## METHODS

### Subjects

Forty-nine individuals took part in the study for financial remuneration or partial course credit and were divided into three tDCS conditions (TPJ: *n* = 17; Sham: *n* = 16; IFC: *n* = 16). The groups were matched in terms of participant gender^2^ (TPJ: 9 female; Sham: 10 female; IFC: 13 female; ${\text{χ}}^{2}$ = 3, *P > *0.2), age (TPJ: *M* = 28.35, s.d. (standard deviation) = 10.26; Sham: *M* = 27.94, *SE* = 7.33; IFC: *M* = 26, s.d. = 10.01; *F* (2, 47) = 0.28, *P > *0.7), handedness (TPJ: 1 left-handed; Sham: 1 left-handed; IFC: 0 left-handed; ${\text{χ}}^{2}$ = 0.04, *P* > 0.9) and electrode impedance during tDCS (TPJ: *M* = 25.91 kΩ, s.d. = 2.00 kΩ; Sham: *M* = 23.92 kΩ, s.d. = 3.45 kΩ; IFC: *M* = 21.20 kΩ, s.d. = 2.65 kΩ; *F* (2, 47) = 0.75, *P > *0.4). All of the participants read a tDCS information sheet and verified that they did not display any contraindications to tDCS. The experiment was approved by the local Research Ethics Committee and was conducted according to the Declaration of Helsinki (1964). One participant in the TPJ condition performed poorly on the imitation inhibition and non-imitative inhibitory control tasks, falling 3.25 s.d. below the mean performance level across conditions—this individual was removed from further analysis, leaving a final sample of 48 participants (i.e. 16 per tDCS condition).

### Stimuli and materials

Stimulation sites for the tDCS protocol were identified using an EasyCap (EasyCap, Herrsching, Germany) landmark cap modified according to standard 10% landmarks. tDCS was delivered through a pair of 35 cm^2^ sponge electrodes, soaked in saline and connected to a neuroConn DC-stimulator Plus (neuroConn, Ilmenau, Germany).

Stimuli for the imitation inhibition and non-imitative inhibitory control tasks were adopted from previous experiments (Figure 1; Brass *et al.*, 2000; Cook and Bird, 2011, 2012; Hogeveen and Obhi, 2013). Stimuli were presented using Superlab v.4.5 (Cedrus Corporation, San Pedro, California) run on a 13″ Macbook Pro laptop (Apple Inc., Cupertino, California, OS X 10.8). During the social interaction task, participants described a set of affectively neutral photographs taken from National Geographic with a confederate.

## PROCEDURES

### tDCS procedures

Previous work implicates primarily right-lateralized TPJ activity (Brass *et al.*, 2005, 2009) and bilateral IFC activity (Brass *et al.*, 2005; Cross *et al.*, 2013) in the control of imitation. Therefore, in this study participants in the TPJ and IFC groups received anodal tDCS to the right hemisphere. The experimenter marked the stimulation sites at FC6 [for IFC stimulation (Holland *et al.*, 2011)] or CP6 [for TPJ stimulation (Santiesteban *et al.*, 2012a)]. Next, the experimenter marked vertex at 50% of the distance between the preauricular points, crossing a point 50% of the distance between inion and nasion. For TPJ stimulation, the anodal electrode was placed at CP6 with the cathodal electrode at vertex, for IFC stimulation the anode was at FC6 with a vertex cathode, and the sham condition was split evenly between the two electrode montages. For the active tDCS conditions, stimulation began with a 15 s ramp-up to 1 mA, proceeded to stimulate at 1 mA for 20 min and ended with a 15 s ramp-down period. For sham stimulation, the same ramping procedure was accompanied by a 30 s stimulation period, yet participants were left in the room for the same total duration, to mimic the experience of real stimulation without any neuromodulatory effect (Gandiga *et al.*, 2006; Nitsche *et al.*, 2008). During the stimulation period, participants were instructed to ‘sit quietly with your eyes closed, think of nothing in particular and let the experimenter know if you experience any discomfort’. These instructions are akin to those used in studies of the default mode network and are designed to minimize any attention to environmental stimuli during stimulation (cf. Damoiseaux *et al.*, 2006; Tambini *et al.*, 2010). Therefore, this study contained 20 min of ‘offline’ tDCS followed by the three behavioral tasks which lasted ∼40 min in total. Previous studies using measures of corticospinal excitability have suggested that the neuromodulatory effects of 13 min of active tDCS are robust for 90 min post-stimulation (Nitsche and Paulus, 2001), suggesting that our protocols were completed within the critical window of the effects of tDCS.

### Imitation inhibition and non-imitative inhibitory control tasks

The imitation inhibition and non-imitative inhibitory control tasks were performed concurrently, either immediately after tDCS, or after the social interaction task, counterbalanced across participants. In both tasks, participants made index or middle finger lifts on a computer keyboard in response to the cue 1 or 2, respectively. At cue onset, an onscreen hand was manipulated in one of the following ways: (i) a congruent or incongruent action was performed (imitation inhibition task; congruent: the action performed by the hand was the same as that which the participant was required to perform, i.e. an index finger lift when an index finger lift response was required; incongruent: the action performed was the opposite to that required of the participant; Brass *et al.*, 2000, 2009), (ii) a congruent or incongruent effector was highlighted (non-imitative inhibitory control task; Cook and Bird, 2011, 2012) or (iii) the image became pixelated (low-level baseline trials; Sowden and Catmur, 2013; Figure 1A). Participants completed 20 trials of each type, split into two randomized blocks of 50 trials.

During the imitation inhibition task participants must inhibit the tendency to imitate on incongruent trials by enhancing their own motor plan and suppressing that of the other. During the non-imitative inhibitory control task participants must inhibit the tendency to move the highlighted finger on incongruent trials, but do not need to control co-activated self- and other-related motor plans. The non-imitative inhibitory control task was designed so that it matched the imitation inhibition task in terms of the irrelevant stimulus dimension’s spatial information and action affordances (Cook and Bird, 2011, 2012). The size of imitation effects on tasks such as those used here have previously been shown to vary as a function of response time (e.g. Press *et al.*, 2005; Catmur and Heyes, 2011), therefore performance on the pixelated baseline condition was regressed out of the data during analysis. The onscreen hand was displayed orthogonal to the orientation of the participant’s hand to reduce the impact of spatial compatibility effects on response times. Furthermore, to prevent any confounding orthogonal spatial compatibility effects, half of the participants in each between-subjects condition performed their responses in the left hemispace and half in the right hemispace (Cho and Proctor, 2004). Response hemispace did not affect performance on any conditions in the imitation inhibition and non-imitative inhibitory control tasks (all *P*s > 0.2) and had no influence on the critical interaction effect reported in the results section (*P* > 0.9).

### Social interaction task

Participants completed the social interaction task with a study confederate. The experimenter told the participants that he was heading to a waiting area to meet a second participant, during which time participants were filmed alone in the room for a period of 1-2 min (*M* = 1.33, s.d. = 0.39 min). The experimenter mentioned the video camera to participants prior to the tDCS procedures, and then covertly started the recording using a remote control just before leaving for the waiting area, to keep participants naive as to which phase(s) of the experiment were being video recorded without deceiving them as to the presence of the camera. Next, the participant and confederate were introduced to each other and seated in chairs placed ∼ 1.5 m apart and at ∼ 45° to each other. The confederate was not aware of the stimulation sites and was not in the room when tDCS was performed—thus, the confederate was sufficiently blind to the predicted effect(s) of tDCS. The experimenter handed each ‘participant’ a set of six miscellaneous photographs and asked them to take turns describing what was in each photograph to the other (Chartrand and Bargh, 1999). The experimenter watched the confederate and participant describe their first photographs and then left the room. For the duration of the interaction (*M* = 12.94, s.d. = 3.68 min), the confederate consistently and unobtrusively touched her face, and the degree to which participants performed this target behavior, or a hand touching control behavior, was coded offline from videos of the interaction.

Two naive observers coded the social interaction videos independently. Specifically, they watched each video and coded the amount of time the participant spent in the room alone (baseline phase) and the amount of time they spent performing the photo description task with the confederate (social interaction phase). Within each phase, coders also tracked the number of times the participant touched their neck or part of their face (target movement), and the number of times they touched their hands together (control movement). The number of times each movement was performed was divided by the corresponding time spent in each phase, providing four movement rates: (i) baseline hand touches per minute, (ii) baseline face touches per minute, (iii) interaction hand touches per minute and (iv) interaction face touches per minute. Further, the number of hand and face touches performed by the confederate during the interaction phase was counted to ensure that the exposure to those movements did not differ between the stimulation conditions (see Results). These behaviors were scored reliably (α = 0.72), therefore all inferential results from the social mimicry task were computed using averaged scores from the two raters.

## RESULTS

### Data preprocessing

Participants performed the social interaction task in one phase of the experiment, and the imitation inhibition and non-imitative inhibitory control tasks were performed concurrently in another phase. The order of these two phases were counterbalanced, but task order did not affect participants’ rate of face touching or hand touching during the social interaction (*P*s > 0.3), nor did it affect performance on the imitation inhibition/non-imitative inhibitory control tasks (*P*s > 0.5). Therefore, order was not included in the main analysis.

Reaction times from the imitation inhibition and non-imitative control tasks were trimmed to remove outliers that were 2.5 s.d. above or below the mean within each experimental condition. Overall accuracy was high in the experiment (*M* = 97%) and to mitigate the influence of any confounding criterion shifts as a function of the tDCS manipulation, inverse efficiency [IE = reaction time/(1—proportion of errors)] scores were computed (Shore *et al.*, 2006; Putzar *et al.*, 2007; Teufel *et al.*, 2010; Hogeveen and Obhi, 2013; Obhi *et al.*, 2014). As expected, all tDCS groups performed significantly better on congruent relative to incongruent trials for both the imitation inhibition (all *P*s < 0.01) and non-imitative inhibitory control (all *P*s < 0.03) tasks. To operationalize ‘imitation inhibition’ and ‘non-imitative inhibitory control’, difference scores between incongruent and congruent trials were computed for both tasks to quantify the size of the inhibition effects. In order to account for any variance driven purely by response speed, our main dependent measure from the imitation inhibition and non-imitative inhibitory control tasks was a standardized residual of a regression of IE difference scores for the two tasks, partialling out performance on the baseline pixelated hand condition (cf. Press *et al.*, 2005; Catmur and Heyes, 2011).

**Fig. 2:**
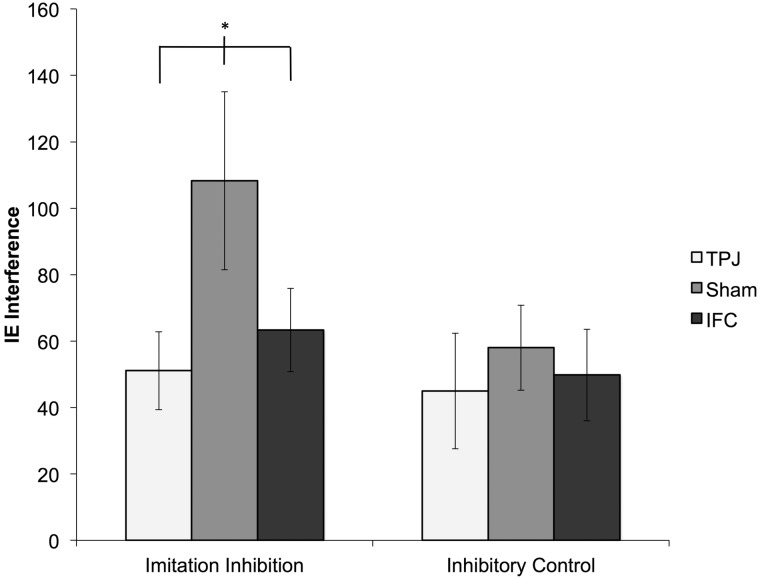
Imitation inhibition was reduced by anodal stimulation of TPJ and IFC relative to sham stimulation while a closely matched inhibitory control task was not. Figure depicts the raw IE effects (i.e. incongruent IE—congruent IE), but in the formal analyzes baseline task performance was also controlled for (*indicates significance at *P* < 0.05).

**Fig. 3:**
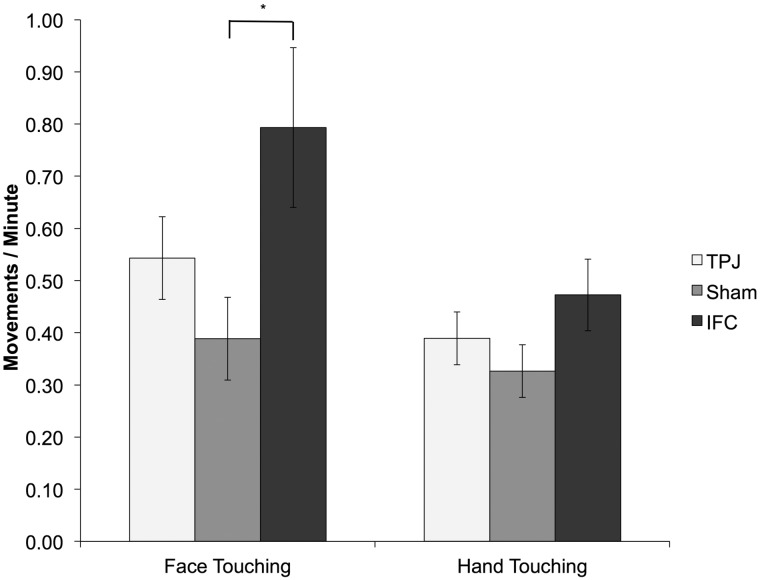
Face touches were performed significantly more in the IFC condition than the Sham condition following observation of face touching. For ease of interpretation, the raw data are depicted, but in the formal analyzes baseline behavior was also controlled for (*represents significance at *P* < 0.05).

Our main dependent measure of mimicry during the social interaction was the standardized residual of a regression of interaction face touches per minute (dependent variable) on baseline face touches per minute (independent variable), and a similar dependent measure was constructed for the control hand touching movement. The use of standardized residuals from all behavioral tasks enabled us to analyze our data within one analysis of variance (ANOVA). However, to aid comparison with previous literature, the data presented in Figures 2 and 3 are in their raw, unstandardized form.

### Main ANOVA

Our main analysis was a 3 (task: imitation inhibition, non-imitative inhibitory control, social interaction) by 3 (stimulation: IFC, TPJ or Sham) mixed model ANOVA. As predicted, the effect of stimulation varied according to task, revealed by a significant interaction between task and stimulation [*F* (4,90) = 2.95, *P* < 0.05, ${\text{η}}_{p^{2}}$ = 0.12]. The between-subjects stimulation factor was not significant (*F* (2,45) < 1, ${\text{η}}_{p^{2}}$ = 0.04). Following the main ANOVA, the significant interaction was broken down by task and type of stimulation through simple effects analyzes.

### Imitation inhibition and non-imitative inhibitory control data

Analysis of the imitation inhibition task revealed a main effect of stimulation [*F* (2,45) = 3.35, *P* < 0.05, ${\text{η}}_{p^{2}}$ = 0.13]. Further simple effects analysis revealed that both TPJ (*P* = 0.04, *d* = 0.58) and IFC (*P* = 0.03, *d* = 0.54) stimulation significantly improved the ability to inhibit imitation compared with Sham. In contrast, performance on the non-imitative inhibitory control task was not affected by either TPJ or IFC stimulation (main effect of stimulation: *F* (2,45) < 1, ${\text{η}}_{p^{2}}$ = 0.02; Figure 2).

### Social interaction data

During the social interaction task, by design, participants were exposed to a larger number of confederate face touches (*M* = 80.13, s.d. = 30.89) than hand-touches [*M* = 28.22, s.d. = 31.90; *t* (47) = 10.91, *P < *0.05, *d* = 1.58]. Importantly, the number of times the target [*F* (2,45) < 1, ${\text{η}}_{p^{2}}$ = 0.02] and control [*F* (2,45) < 1, ${\text{η}}_{p^{2}}$ < 0.01] movements were performed by the confederate was consistent across tDCS conditions.

Analysis of face touching behavior during the interaction revealed a significant effect of stimulation [*F* (2,45) = 3.2, *P < *0.05, ${\text{η}}_{p^{2}}$ = 0.12]. Simple effects analyzes revealed that this was due to the fact that, while stimulation of IFC (*P* = 0.02, *d* = 0.62) increased the degree of imitation during the task relative to sham, TPJ stimulation did not (*P* = 0.43, *d* = 0.20). The rate of the control movement, hand touching, did not vary as a function of stimulation [*F* (2,45) < 1, ${\text{η}}_{p^{2}}$ = 0.03; Figure 3].

## DISCUSSION

Successful social interaction requires rapid control of socio-cognitive processes. Perhaps the best studied of these processes is the tendency to imitate (Heyes, 2011; Chartrand and Lakin, 2013). Humans modulate the degree to which they imitate each other with exquisite precision in order to produce high-quality social interaction (Lafrance and Broadbent, 1976; Chartrand and Bargh, 1999; Lakin and Chartrand, 2003; Neal and Chartrand, 2011; Chartrand and Lakin, 2013). Through the use of tDCS to enhance cortical excitability at IFC and TPJ, we demonstrate that the imitative control functions of these two regions are distinct.

Enhancing IFC excitability produced opposite effects depending on task requirements. During the imitation inhibition task, stimulation of IFC resulted in a greater ability to inhibit imitation, leading to improved task performance. Conversely, during the social interaction task—in which greater imitation is associated with better social interaction—stimulation of IFC increased the degree of imitation exhibited by participants. This enhanced control of imitation through IFC stimulation was specific to imitation; it was not seen in a closely matched inhibitory control task nor in non-imitative movements during social interaction. The present results complement the findings of enhanced IFC activation during both the performance (e.g. Iacoboni *et al.*, 1999) and inhibition (e.g. Brass *et al.*, 2005) of imitation, suggesting that the effect of tDCS to IFC was not simply enhanced mirroring or enhanced inhibition, but enhanced control of the mirror system’s impact on overt behavior. Together with the previous neuroimaging studies, these data support a model of imitative control in which IFC serves either to inhibit or to enhance imitation, depending on task demands, and where this control is distinct from control of other automatic behavioral tendencies (Brass *et al.*, 2005, 2009; Spengler *et al.*, 2010b; Santiesteban *et al.*, 2012a; Sowden and Catmur, 2013).

It is worth considering an alternative explanation of the IFC data in which stimulation excited two distinct populations of neurons; one involved in action mirroring and the other involved in response inhibition. The spatially diffuse nature of tDCS makes it likely that multiple populations of neurons within the IFC are excited—and it is possible that the mirror population of neurons is recruited during the social interaction task while the pool of inhibition neurons is recruited during the imitation inhibition task. However, the fact that there was no effect of stimulation on the non-imitative inhibitory control task suggests that this ‘two-population’ explanation is less likely to explain the IFC data than a specific effect on the control of imitation. Regardless, this issue is worthy of further investigation using a spatially more precise technique such as transcranial magnetic stimulation (TMS).

The high degree of selectivity of the IFC stimulation effect—impacting upon the imitation inhibition task but not the non-imitative inhibitory control task—is surprising, especially given the role of the IFC in inhibition more generally (e.g. Aron *et al.*, 2004). However, such selectivity is in line with recent results reported by Sowden and Catmur (2013) who showed that applying repetitive TMS to TPJ impaired imitation inhibition but did not modulate spatial compatibility effects. The current data therefore complement by Sowden and Catmur’s (2013) work, suggesting that domain-general inhibitory control systems and domain-specific imitation-inhibition systems are at least partially distinct.

TPJ stimulation also showed task-dependent effects on performance, but these were different from those observed under IFC stimulation. Excitation of TPJ resulted in an increased ability to inhibit imitation but did not affect performance on the social mimicry task nor on the non-imitative inhibitory control task. The effect of stimulation of TPJ supports the suggestion that this area is involved in the online control of representations of the self and others. Across the three tasks used in this study it was only the imitation inhibition task in which participants were required to differentiate and control co-activated motor representations according to whether they were a result of the participant’s own motor plan or whether they were prompted by another. The effect of TPJ stimulation is in accordance with the results of Santiesteban *et al.* (2012a) who showed an effect of TPJ stimulation on imitation inhibition and also another task requiring control of self-other representations, visual perspective taking. However, the present data go beyond those of Santiesteban *et al.* (2012a) in two ways. First, they demonstrate an effect of TPJ stimulation that is specific to self-other control. The lack of an effect of stimulation on the non-imitative inhibitory control task is especially striking, as the task instructions and responses were identical to those of the imitation inhibition task. Second, they demonstrate how the different components of the imitation control network contribute to the control of social cognition as a function of task demands.

As a final consideration, the active stimulation conditions included a cathodal electrode placed over Cz, extending anteriorly over primary motor cortex (M1). Because cathodal stimulation reduces cortical excitability at structures underlying the electrode (Nitsche *et al.*, 2003), and this electrode might have reduced corticospinal excitability in the present experiment. As many researchers now consider M1 to be part of the broader MNS (Tkach *et al.*, 2007), and the role of the MNS in imitation is well known (Heiser *et al.*, 2003; Catmur *et al.*, 2009), the cathodal stimulation might have been expected to reduce the degree of imitation observed in this study. However, results were unlikely to be a product of cathodal stimulation as the Cz electrode position means that any stimulation effect would be on the dorsal-most portion of M1, impacting upon more distal body sites (e.g. legs, hips, trunk) than the proximal effectors involved in the two imitation tasks (i.e. hands/arms in the social interaction task, and the index/middle fingers in the imitation-inhibition task). Furthermore, cathodal stimulation of vertex was consistent across the TPJ and IFC groups and yet anodal stimulation of these areas produced dissociable effects. Although these factors do not rule-out important effects of stimulation at the vertex, they suggest that the stimulation effects observed in this study are unlikely to be a product of cathodal stimulation.

Until recently this area of social neuroscience has received little attention, despite several studies demonstrating the importance of social control processes for interaction quality. Understanding the way in which socio-cognitive processes are controlled at the neural level, and how they may develop atypically in disorders characterized by poor social interaction such as ASDs, is therefore of vital importance.

## Conflict of Interest

None declared.

## Footnotes

^1^Extant neuroimaging data suggests that IFC, referring to posterior portions of the inferior frontal gyrus and ventral premotor cortex, contains populations of cells with both facilitative mirror properties—i.e. that match observed and executed representations of action (Iacoboni *et al.*, 1999; Kilner *et al.*, 2009; Press *et al.*, 2012a,b)—and also a population of cells involved in the control of the mirror system, which is active when the mirror system must be inhibited to avoid task-inappropriate imitation (Brass *et al.*, 2005, 2009; Cross *et al.*, 2013)

^2^Although gender balance was statistically matched between groups, the IFC stimulation group contained a numerically larger number of females than the other groups. To ensure that results were not due to this imbalance, results were re-analyzed including a covariate coding for gender. The inclusion of the gender covariate did not change the pattern of significance and therefore the results presented below are from an analysis excluding this covariate.
